# An improved genetic system for bioengineering buoyant gas vesicle nanoparticles from Haloarchaea

**DOI:** 10.1186/1472-6750-13-112

**Published:** 2013-12-21

**Authors:** Shiladitya DasSarma, Ram Karan, Priya DasSarma, Susan Barnes, Folasade Ekulona, Barbara Smith

**Affiliations:** 1 Institute of Marine and Environmental Technology and Department of Microbiology and Immunology, University of Maryland School of Medicine, 701 E Pratt Street, Baltimore, MD 21202, USA; 2 Johns Hopkins School of Medicine Microscope Facility, Baltimore, MD 21205, USA

**Keywords:** Vaccine, Halophiles, Archaea, Luciferase

## Abstract

**Background:**

Gas vesicles are hollow, buoyant organelles bounded by a thin and extremely stable protein membrane. They are coded by a cluster of *gvp* genes in the halophilic archaeon, *Halobacterium* sp. NRC-1. Using an expression vector containing the entire *gvp* gene cluster, gas vesicle nanoparticles (GVNPs) have been successfully bioengineered for antigen display by constructing gene fusions between the *gvp*C gene and coding sequences from bacterial and viral pathogens.

**Results:**

To improve and streamline the genetic system for bioengineering of GVNPs, we first constructed a strain of *Halobacterium* sp. NRC-1 deleted solely for the *gvp*C gene. The deleted strain contained smaller, more spindle-shaped nanoparticles observable by transmission electron microscopy, confirming a shape-determining role for GvpC in gas vesicle biogenesis. Next, we constructed expression plasmids containing N-terminal coding portions or the complete *gvp*C gene. After introducing the expression plasmids into the *Halobacterium* sp. NRC-1 Δ*gvp*C strain, GvpC protein and variants were localized to the GVNPs by Western blotting analysis and their effects on increasing the size and shape of nanoparticles established by electron microscopy. Finally, a synthetic gene coding for *Gaussia princeps* luciferase was fused to the *gvp*C gene fragments on expression plasmids, resulting in an enzymatically active GvpC-luciferase fusion protein bound to the buoyant nanoparticles from *Halobacterium*.

**Conclusion:**

GvpC protein and its N-terminal fragments expressed from plasmid constructs complemented a *Halobacterium* sp. NRC-1 Δ*gvp*C strain and bound to buoyant GVNPs. Fusion of the luciferase reporter gene from *Gaussia princeps* to the *gvp*C gene derivatives in expression plasmids produced GVNPs with enzymatically active luciferase bound. These results establish a significantly improved genetic system for displaying foreign proteins on *Halobacterium* gas vesicles and extend the bioengineering potential of these novel nanoparticles to catalytically active enzymes.

## Background

Buoyant gas vesicles are prokaryotic organelles that are widely distributed among bacterial and archaeal microorganisms and constitute protein nanoparticles (GVNPs) that may be engineered for biotechnological applications [1-3]. These organelles naturally promote flotation and increase the availability of light and oxygen to many aquatic microorganisms, especially those with photosynthetic or phototrophic capabilities. Water is excluded from the interior, a property that is thought to be a consequence of the hydrophobicity of the interior surface of the proteinaceous membrane. While the exact protein composition of the membrane has been difficult to ascertain due to its extreme stability against solubilization, production of these structures is easily scaled-up and they are simple to purify by hypotonic lysis of the host and concentrate by flotation, enhancing their intrinsic value for biotechnological applications [4,5].

**Figure 1:**
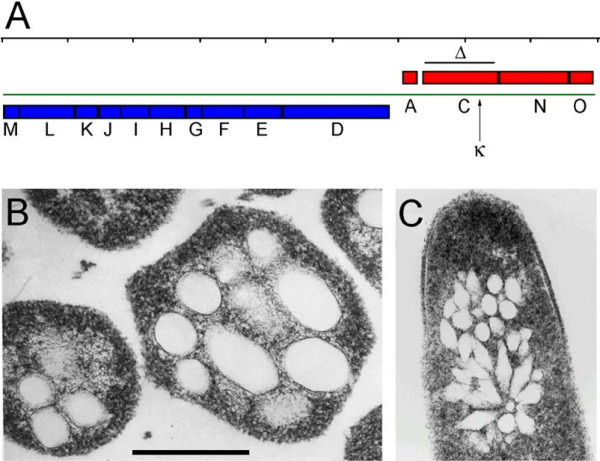
***Halobacterium *sp. gas vesicle gene cluster and thin-sections. (A)** Genetic map of the gas vesicle gene cluster from *Halobacterium* sp. NRC-1 pNRC100 is shown with genes transcribed rightward colored red and genes transcribed leftward colored blue [14]. The scale is noted above (divided into kilobase pairs) and the positions of the *gvp*C deletion (Δ) and kappa insertion (κ) are indicated. **(B)** and **(C)** Thin sections of *Halobacterium* sp. NRC-1 **(B)** and SD109 (pFM104*gvp*C::κ1) mutant **(C)** viewed by transmission electron microscopy (bar, which is 325 nm long, applies to both **B** and **C**). Gas vesicles are visible as empty oval or spindle-shaped regions. Shapes observed reflect different planes of sectioning.

Genetic analysis established the importance of a gene cluster (*gvp*MLKJIHGFEDACNO) for gas vesicle formation in large plasmids of extremely halophilic Archaea (Haloarchaea) (Figure 1A) [6-10]. In *Halobacterium* sp. NRC-1, the gene cluster was found on a 191 kb plasmid, pNRC100, with transcription of *gvp*ACNO oriented rightward, transcription of *gvp*DEFGHIJKLM oriented leftward, and divergent promoters located in the 201 bp *gvp*A-D intergenic region. Mutants constructed with interruptions in each of the *gvp* genes by a kanamycin cassette (κ) exhibited a partially or completely gas vesicle-deficient phenotype, indicating that all of the *gvp* genes are necessary for wild-type gas vesicle formation [11]. This genetic system utilized the natural gas vesicle-deficient mutant strain SD109, with a complete deletion of the *gvp* gene cluster, and pFM104d, a large (18.9 kbp) *Halobacterium*-*E. coli* shuttle plasmid containing the entire 8.9 kbp *gvp* gene cluster [4,5,11-14].

The protein composition of gas vesicle nanoparticles has been studied primarily by Western blotting analysis using antisera directed against individual *gvp* gene products [15]. Initially, only GvpA and GvpC proteins were found [8], but further analysis showed the presence of five additional proteins, GvpF, GvpG, GvpJ, GvpL, and GvpM [15]. GvpA, J, and M constitute a small family of proteins (Pfam 741) likely involved in gas vesicle membrane formation, while GvpF and L are coiled-coil proteins (Pfam 6386) with self-associative properties thought to be important for nucleation or growth of the nanoparticles [9,15]. Most of these proteins (GvpA, GvpC, GvpF, GvpJ, and GvpL) were also identified in a recent proteomic study [16]. In genome sequencing studies, genes corresponding to these same proteins were also found in other gas vesicle-forming microbes [17]. An exception was the *gvp*C gene, which was reported only in the haloarchaeal and cyanobacterial gas vesicle producers.

In *Halobacterium* sp. NRC-1, the *gvp*C gene encodes a hydrophilic protein with a predicted molecular weight of 42,391 and a highly acidic pI of only 3.57 [8,9]. In this haloarchaeon, the GvpC protein sequence contains 8 imperfect repeats and an extremely acidic stretch located near the C-terminus. The slight similarity of the haloarchaeal repeats to the repeats in the cyanobacteria suggested that the GvpC proteins play similar roles in both haloarchaea and cyanobacteria [18]. In the cyanobacterium, *Anabaena flos-aquae*, GvpC has been shown to serve a strengthening role in gas vesicles [19], while in *Halobacterium* sp. NRC-1, insertion mutations in the *gvpC* gene generated vesicles with altered shape and size [11]. These findings suggested that GvpC proteins facilitate gas vesicles’ growth and enhance stability in strains which produce them.

The potential value of GvpC protein for bioengineering floating GVNPs was established during mutagenesis of the *gvp* gene cluster from *Halobacterium* sp. NRC-1. A *gvp*C::κ insertion mutant was found to produce primarily spindle-shaped gas vesicles with smaller than wild-type size (Figure 1B & C) and excision of most of the κ insert resulted in the production of vesicles with a peptide fused to GvpC protein that was antigenically displayed and immunologically accessible on the surface [7,11]. Further studies with SIV and chlamydial proteins have shown that bioengineered GVNPs may be used for antigen display and elicit both humoral and cellular responses in mice [4,5,20-22].

The genetic system currently in use for bioengineering of gas vesicle nanoparticles is technically challenging due to the large size and complexity of the *gvp* gene cluster [7,8]. In order to facilitate bioengineering of nanoparticles, we constructed a new *Halobacterium* sp. NRC-1 derived host strain and a series of smaller, more versatile plasmid expression vectors. The work documented in this report establishes a significantly improved genetic system for expression of GvpC-fusion proteins, including an active luciferase enzyme from *Gaussia princeps*[23].

## Results

### Construction of a *Halobacterium* Δ*gvp*C strain and *gvpC* expression vectors

In order to improve the genetic system for bioengineering of GVNPs [7,8], our first goal was the construction of a *gvp*C deletion strain, via the *ura*3-based gene deletion method for *Halobacterium* sp. NRC-1 [24,25]. Approximately 500-bp flanking regions of *gvp*C, including the first and last few codons of *gvp*C, were cloned into the suicide vector, pBB400 [26], and the resulting plasmid, pBB400Δ*gvp*C, was used to transform *Halobacterium* sp. NRC-1Δ*ura*3. After selecting sequentially for integration and excision (see Methods), the resulting *Halobacterium* sp. NRC-1Δ*ura*3Δ*gvp*C deletion strain (referred henceforth as Δ*gvp*C deletion strain) (Figure 1A) showed a partially gas vesicle-deficient phenotype with small, largely spindle-shaped gas vesicles, similar to that reported for a *gvp*C::κ insertion mutant (cf. Figure 1B and C).

**Figure 2:**
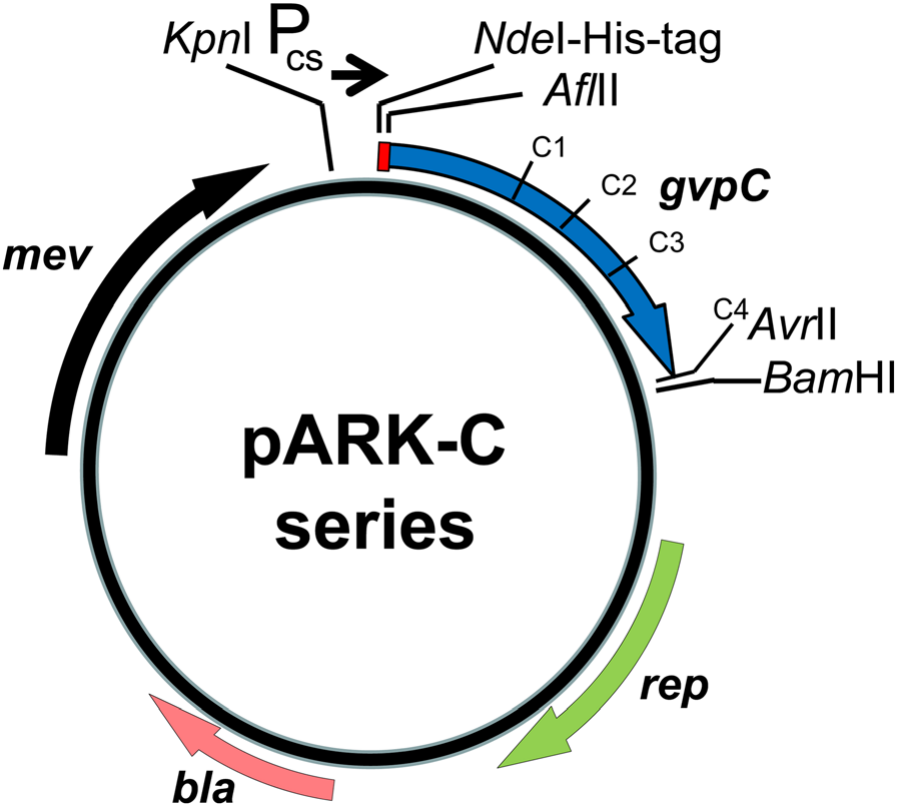
**Structure of pARK-C plasmid series used for expression of *gvp*C fragments in *Halobacterium *sp. NRC-1 and ∆ *gvp *C strains.** Location and transcriptional orientation of *bla* (pink), β-lactamase, for ampicillin resistance; *mev* (black), HMG-CoA reductase for mevinolin resistance; C1-C4, *gvp*C segments (blue), and *rep* (green), the *Halobacterium* pGRB replicase gene are shown. Position of the temperature-induced *csp*D2 promoter (labeled P_cs_ →), His-tag (red box), and *Kpn*I, *Nde*I, *Afl*II, *Avr*II, and *Bam*HI restriction sites are indicated.

To further develop the expression system and test for complementation of the Δ*gvp*C strain, we constructed a *gvp*C expression vector series (Figure 2). As the backbone, we used pMC2, an expression plasmid with the high-level cold-inducible *csp*D2 promoter, and the ability for replication and selection in both *E. coli* and *Halobacterium*, recently constructed for investigation of a β-galactosidase protein from a related haloarchaeon [27]. The β-galactosidase gene was excised and replaced with an adapter containing a start codon, a hexahistidine-tag (His-tag), and unique restriction sites for insertion of the foreign genes, resulting in the plasmid pARK (Table 1). A series of PCR amplified *gvp*C gene fragments were then inserted into the unique *Afl*II and *Avr*II sites in the adapter region of pARK to construct the pARK-C plasmid series containing various regions of *gvp*C (Figure 2).

**Table 1:** Strains and plasmids used in this study

**Strain/plasmid**	**Characteristics**	**Source or reference**
*Halobacterium* sp. strain NRC-1	Sequenced wild-type strain	Laboratory collection [28]
*Halobacterium* sp. strain SD109	Strain NRC-1 deleted for the *gvp* gene cluster of pNRC100	Laboratory collection [13]
*Halobacterium* sp. strain NRC-1Δ*ura*3	Strain NRC-1 deleted for *ura*3 gene coding orotidine-5′-monophosphate	Laboratory collection [24-27]
*Halobacterium* sp. strain NRC-1Δ*ura*3Δ*gvp*C	Strain NRC-1Δ*ura*3 deleted for the *gvp*C gene	This study
pFM104*gvp*C::κ1	*Halobacterium-E. coli* shuttle plasmid containing entire *gvp* gene cluster with κ insertion in the *gvp*C gene	Laboratory collection [11]
pBB400	Suicide plasmid capable of replication in *E. coli* but not *Halobacterium,* containing the *ura*3 gene	Laboratory collection [26]
pBB400Δ*gvp*C	pBB400 plasmid containing *gvp*C gene-flanking regions for deletion construction	This study
pMC2	*Halobacterium* expression vector with *csp*D2 promoter and *Halorubrum lacusprofundi* β-galactosidase gene	Laboratory collection [27]
pARK	*Halobacterium* sp. NRC-1 expression vector with *csp*D2 promoter and adapter containing a start codon, His-tag, and restriction sites for insertion of the *gvp*C gene fragments	This study
pARK-C1	pARK derivative with *gvp*C gene C1 fragment	This study
pARK-C2	pARK derivative with *gvp*C gene C2 fragment	This study
pARK-C3	pARK derivative with *gvp*C gene C3 fragment	This study
pARK-C4	pARK derivative with *gvp*C complete gene (C4)	This study
pDRK	*Halobacterium* sp. NRC-1 expression vector with *gvp*A promoter and adapter containing a start codon, His-tag, and restriction sites used for insertion of the *gvp*C gene fragments	This study
pDRK-C1-L	pDRK derivative containing *gvp*C gene C1 fragment fused to codon-optimized *Gaussia princeps* luciferase gene	This study
pDRK-C2-L	pDRK derivative containing *gvp*C gene C2 fragment fused to codon-optimized *Gaussia princeps* luciferase gene	This study
pDRK-C3-L	pDRK derivative containing *gvp*C gene C3 fragment fused to codon-optimized *Gaussia princeps* luciferase gene	This study
pDRK-C4-L	pDRK derivative containing *gvp*C complete gene (C4) fused to codon-optimized *Gaussia princeps* luciferase gene	This study

### Engineering of the *gvp*C gene and expression of GvpC fragments

**Figure 3:**
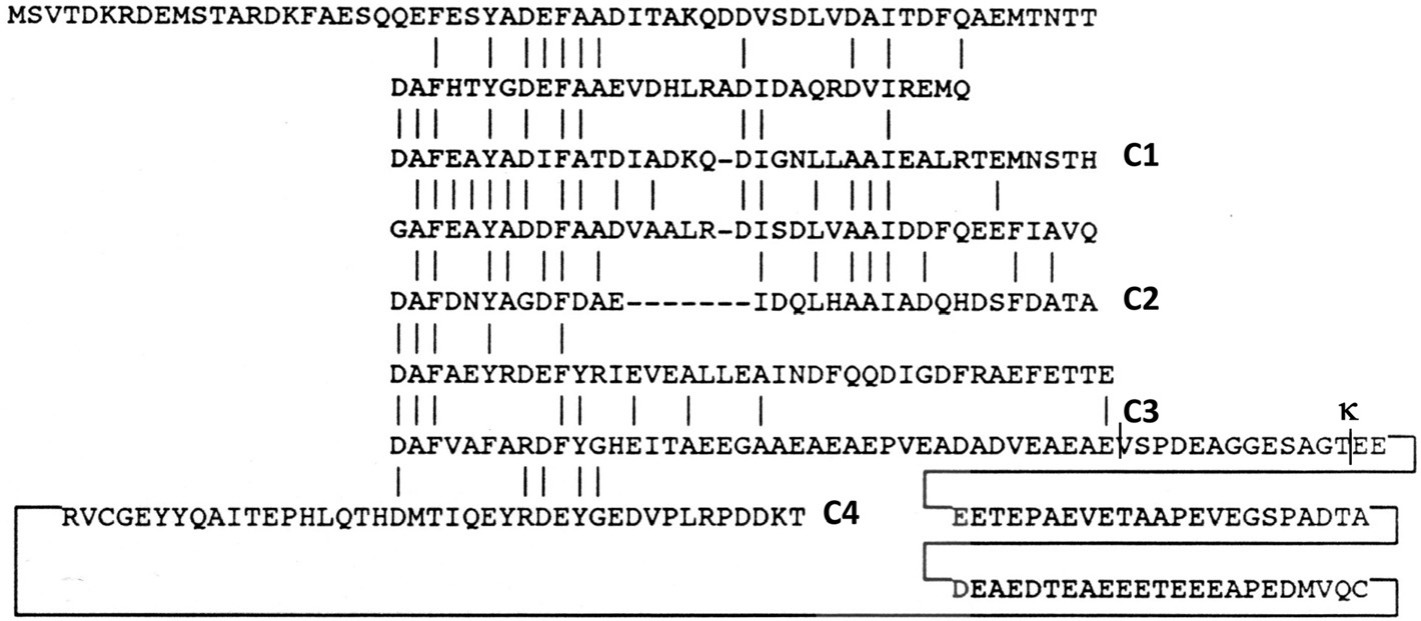
**GvpC protein sequence and engineering sites.** The amino acid sequence of GvpC of *Halobacterium* sp. NRC-1 is shown with conserved residues (vertical bars) in the eight imperfect repeats. The GvpC segments used in this study are labeled C1, C2, C3 and C4 at the C-terminal end. The position of the κ insertion in the *gvp*C mutant is labeled ‘κ’.

**Figure 4:**
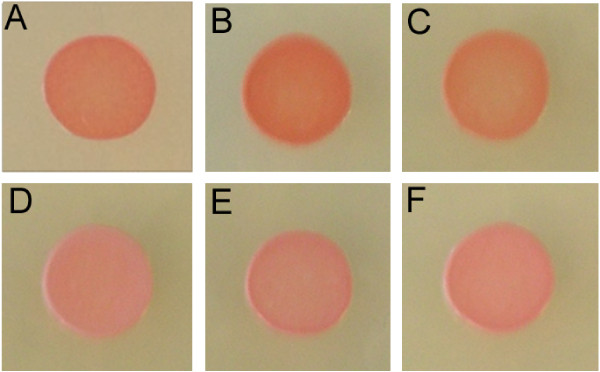
**Phenotype of *Halobacterium *sp. NRC-1∆*ura*3∆*gvp*C and derivative strains containing *gvp*C expression plasmids with fragments of varying lengths.** Increasing opacity (from orange to pink color) indicates higher levels of GVNPs produced in cells. Cultures of the following strains are shown spotted on CM^+^ plates: **A**: *Halobacterium* sp. NRC-1Δ*ura*3Δ*gvp*C, **B**: *Halobacterium* sp. NRC-1Δ*ura*3Δ*gvp*C (pARK-C1), **C**: *Halobacterium* sp. NRC-1Δ*ura*3Δ*gvp*C (pARK-C2), **D**: *Halobacterium* sp. NRC-1Δ*ura*3Δ*gvp*C (pARK-C3), **E**: *Halobacterium* sp. NRC-1Δ*ura*3Δ*gvp*C (pARK-C4), and **F**: *Halobacterium* sp. NRC-1Δ*ura*3.

To determine the effect of GvpC length on production and bioengineering of GVNPs, we transformed each member of the pARK-C plasmid series coding N-terminal regions of GvpC into the Δ*gvp*C strain. pARK-C1 contained 130 amino acids of GvpC, pARK-C2 contained 200 amino acids, pARK-C3 contained 280 amino acids, and pARK-C4 plasmid and the wild-type strain contained the complete 382 amino acid GvpC sequence (Figure 3). When the phenotype of the Δ*gvp*C (pARK-C) transformants were compared to the parental Δ*gvp*C and ∆*ura*3 strains, increasing opacity resulting from increasing gas vesicle content was observed in the following order: Δ*gvp*C < Δ*gvp*C (pARK-C1) < Δ*gvp* (pARK-C2) < Δ*gvp*C (pARK-C3) ≈ Δ*gvp*C (pARK-C4) ≈ ∆*ura*3 (Figure 4, cf. panels A-F, respectively). These findings indicated lower quantities of gas vesicle formation in strains expressing smaller fragments of GvpC and higher quantities in strains expressing larger GvpC fragments or the complete GvpC protein.

**Figure 5:**
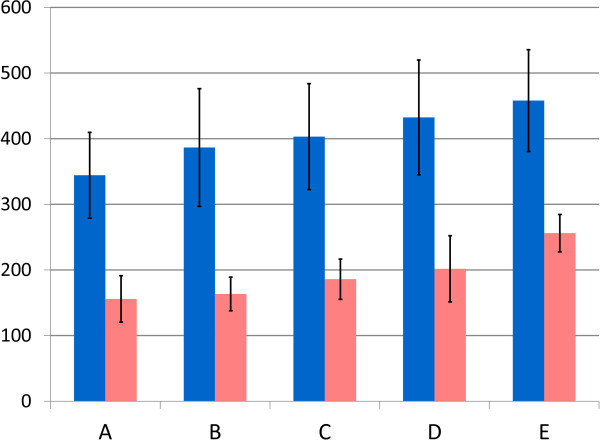
**Gas vesicle nanoparticle dimensions in *Halobacterium *sp. NRC-1∆*ura*3 and ∆*gvp*C derivative strains containing *gvp*C expression plasmids.** Average dimensions for nanoparticle length (blue) and width (pink) are shown in nanometers (vertical axis), and standard deviation shown with error bars. **A**: *Halobacterium* sp. NRC-1Δ*ura*3Δ*gvp*C, **B**: *Halobacterium* sp. NRC-1Δ*ura*3Δ*gvp*C (pARK-C1), **C**: *Halobacterium* sp. NRC-1Δ*ura*3Δ*gvp*C (pARK-C2), **D**: *Halobacterium* sp. NRC-1Δ*ura*3Δ*gvp*C (pARK-C3), **E**: *Halobacterium* sp. NRC-1Δ*ura*3.

To examine the morphology of GVNPs in Δ*gvp*C and derivative Δ*gvp*C (pARK-C series) strains, we treated cells to hypotonic conditions for lysis and purified nanoparticles by centrifugally accelerated flotation (see Methods). The vesicle preparations were spread for electron microscopy and stained with uranyl acetate, and observed as either spindle-shaped or cylindrical-shaped structures. A representative number of GVNPs were measured, confirming that nanoparticles from the parental Δ*gvp*C strain were smaller and more spindle-shaped, as previously observed for the *gvp*C::κ mutant, compared to the parental Δ*ura*3 strain. The mean length and width of GVNPs (blue and pink bars, respectively, in Figure 5) were proportional to the size of *gvp*C gene fragment in pARK-C1, C2, and C3 (386–432 nm lengths and 163–202 nm widths), and were intermediate compared to the Δ*gvp*C (344 and 158 nm, respectively) and Δ*ura*3 (458 and 256 nm, respectively) strains. Expression of larger GvpC proteins generally resulted in production of longer and wider gas vesicles.

**Figure 6:**
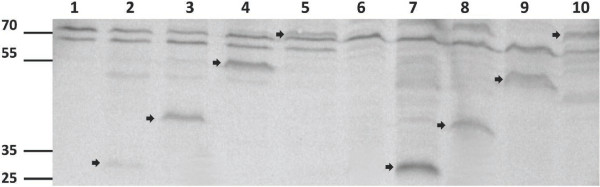
**Western blotting analysis of *Halobacterium *sp. NRC-1∆*ura*3∆*gvp*C and derivative strains containing *gvp*C expression plasmids.** Cell lysates (lanes 1–5) or gas vesicles (lanes 6–10) were electrophoresed on a 12% polyacrylamide-SDS gel, transferred to PVDF membrane, and probed with His-tag antibody followed by secondary antibody-alkaline phosphatase conjugate. Lanes 1 & 6: *Halobacterium* sp. NRC-1Δ*ura*3Δ*gvp*C, Lanes 2 & 7: *Halobacterium* sp. NRC-1Δ*ura*3Δ*gvp*C (pARK-C1), Lanes 3 & 8: *Halobacterium* sp. NRC-1Δ*ura*3Δ*gvp*C (pARK-C2), Lanes 4 & 9: *Halobacterium* sp. NRC-1Δ*ura*3Δ*gvp*C (pARK-C3), Lanes 5 & 10: *Halobacterium* sp. NRC-1Δ*ura*3Δ*gvp*C (pARK-C4).

Western blotting analysis was used to localize GvpC protein and N-terminal fragments expressed from the pARK-C plasmid series in Δ*gvp*C transformants. For this analysis, confluent lawns of cells were grown and lysed by hypotonic lysis. Gas vesicle nanoparticles were collected after low-speed centrifugally accelerated flotation and electrophoresed on SDS-polyacrylamide gels. After transfer, rabbit antisera directed against either the His-tag incorporated at the N-terminal region of GvpC (Figure 6), or a synthetic GvpC peptide (data not shown), were used in Western blotting analysis. The Western blots showed GvpC proteins of expected sizes (N-terminal fragments and full-length GvpC) localized to cell lysates (Figure 6, arrows, lanes 2–5) and floating gas vesicle nanoparticles (Figure 6, arrows, lanes 7–10). As previously reported for the GvpC protein, the apparent protein sizes were significantly larger than predicted from molecular weight standards, due to its high acidity [8,15].

### Luciferase expression and display on gas vesicles

**Figure 7:**
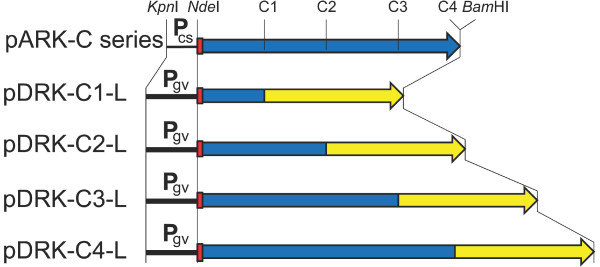
**Partial map of pDRK-C-L plasmid series used for expression of *gvp*C-luciferase fusion genes in *Halobacterium *sp. NRC-1 and ∆*gvp*C strains.** The upper map (pARK-C series) displays the *Kpn*I-*Bam*HI region of the pARK-C series plasmids, with the *csp*D2 promoter labeled P_cs_, His-tag shown as red box, and *gvp*C gene (with C1, C2, C3, and C4 regions marked) shown as blue arrow. The four lower maps (labeled pDRK-C1-L to C4-L) show the *Kpn*I-*Bam*HI regions of pDRK-C-L plasmid series containing the *gvp*A promoter (labeled P_gv_), His-tag (red box), C1, C2, C3, and C4 regions of *gvp*C (blue boxes), and codon optimized *Gaussia princeps* luciferase gene (yellow arrow). The corresponding sites of *Kpn*I, *Nde*I, and *Bam*HI cleavage are indicated, while the *Afe*I sites at the GvpC-luciferase gene boundaries are not shown.

**Figure 8:**
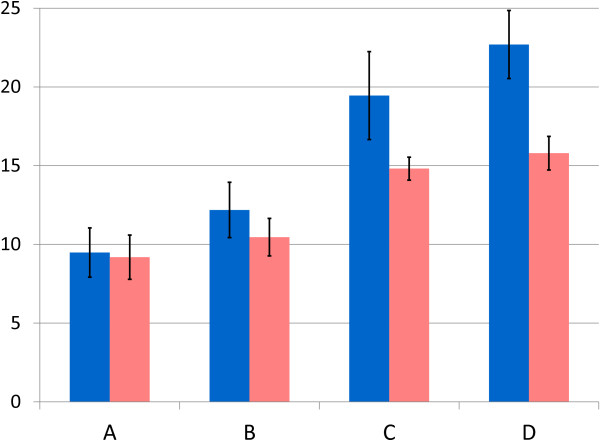
**Luciferase activity in purified GVNPs from *Halobacterium *sp. NRC-1∆*ura*3∆*gvp*C and *Halobacterium *sp. NRC-1 strains containing *gvp*C and luciferase expression plasmids.** Percent luciferase activity, chemiluminescence activity detected in gas vesicles compared to the total activity observed in cell lysates, is plotted on vertical axis for pDRK-C-L plasmid series. Values plotted are the average of experiments performed in triplicate, and standard deviation shown with error bars. **A**: pDRK-C1-L, **B**: pDRK-C2-L, **C**: pDRK-C3-L, **D**: pDRK-C4-L in either *Halobacterium* sp. NRC-1Δ*ura*3Δ*gvp*C (blue), or in wild-type *Halobacterium* sp. NRC-1 (pink) strain.

In order to determine whether GVNPs produced in the Δ*gvp*C strain can be bioengineered to display foreign proteins, we expressed a synthetic luciferase gene from the marine copepod *Gaussia princeps* fused to the *gvp*C gene or its N-terminal fragments [23]. Codons in the synthetic luciferase gene were optimized to reflect usage in *Halobacterium* sp. NRC-1 [29] and the gene was inserted into the pARK-C plasmid series via an engineered *Afe*I site to produce GvpC-luciferase gene fusions (Figure 7). In order to increase the level of expression, the *csp*D2 promoter was replaced by the stronger *gvp*A promoter [9,10,30], recently used to bioengineer extremely radiation resistant derivatives of *Halobacterium* sp. NRC-1 [31], via the *Kpn*I and *Nde*I sites (see also Figure 2 and Table 1). Each member of the constructed plasmid series, named pDRK-C1-L to pDRK-C4-L (Figure 7), was then transformed into both wild-type NRC-1 and Δ*gvp*C strains, transformants were grown as lawns on agar plates and lysed hypotonically, and the GVNPs purified by centrifugally accelerated flotation. To determine whether luciferase was bound to the floating GVNPs, chemiluminescence activity was compared between the subnatant and the floating GVNP fraction. The results showed that GvpC-luciferase fusion protein was bound to GVNPs and was enzymatically active (Figure 8). Higher levels of activity were observed for the fusion proteins with longer GvpC-fragments or the entire GvpC protein (C3 and C4) compared to the shorter fragments (C1 and C2) (cf. C and D versus A and B in Figure 8).

Interestingly, when members of the pDRK-C-L plasmid series were transformed into *Halobacterium* sp. NRC-1, which contains a wild-type *gvp*C gene, nanoparticles containing engineered GvpC-luciferase proteins were also detectable by luciferase activity (Figure 8, pink bars) and Western blotting assays (data not shown). Although higher levels of luciferase activity were observed bound to floating gas vesicle nanoparticles in the Δ*gvp*C (pDRK-C1-L to C4-L) strains compared to the NRC-1 (pDRK-C1-L to C4-L) strains, luciferase activity was clearly measurable in nanoparticles in the transformed wild-type strain (Figure 8). Moreover, the wild-type GvpC protein was also detected bound to GVNPs in the NRC-1 (pDRK-C1-L to C4-L) strains (data not shown), indicating that two different GvpC forms may be simultaneously bound to the nanoparticles. These results extend the possible biotechnological uses of GVNPs to other applications requiring nanoparticle-bound enzymes and multivalency.

## Discussion

We have established an improved genetic system for bioengineering of GVNPs in the model halophilic archaeon, *Halobacterium* sp. NRC-1. A strain deleted for the *gvp*C gene and plasmid vectors containing highly active promoters for producing GvpC-fusion proteins were constructed. The system was tested by expressing the entire *gvp*C gene, N-terminal portions of *gvp*C gene fragments, and GvpC-luciferase fusion proteins, all of which bound to the buoyant nanoparticles. The improved genetic engineering system provides the opportunity for insertion of multiple foreign sequences and the potential for production of GVNPs displaying multiple antigens. The work reported here represents a significant step forward in demonstrating the bioengineering capabilities of GVNPs, including their application to antigen display and vaccine development.

The current work has capitalized on the *Halobacterium* sp. NRC-1 genetic system and recently constructed expression plasmids [25-27,31]. These biotechnological tools have been used to overexpress, purify, and characterize a polyextremophilic β-galactosidase enzyme from an Antarctic haloarchaeon, and bioengineer resistance of haloarchaeal cells to ionizing radiation by overexpression of a mammalian-type RPA protein [27,31]. The constructed expression plasmids (pARK and pDRK) contain the high-copy number *Halobacterium* sp. pGRB miniplasmid for replication and the mevinolin resistance gene for selection in haloarchaea, as well as the plasmid pUC18 vector for replication and selection in the *E. coli* host. The pARK expression plasmids contain the *csp*D2 promoter while the pDRK expression plasmids contain the *gvp*A promoter. Both of these promoters were reported to drive expression of genes inducible under cold temperatures [30]. The pARK and pDRK plasmids were tailored for expression of GvpC fusion proteins and represent convenient vectors for production of bioengineered GVNPs.

Our GVNP-bioengineering and expression system exploits genetic properties of the *Halobacterium* sp. NRC-1Δ*gvp*C deletion strain and recombinant capabilities of the *gvp*C gene from *Halobacterium* sp. NRC-1 pNRC100 plasmid [8,9]. The Δ*gvp*C deletion strain, constructed using our *ura*3-deletion method [24,25], contained gas vesicles with smaller, more spindle-shaped vesicles observable by transmission electron microscopy. This finding is consistent with earlier observations suggesting a key role for GvpC protein in shape determination of gas vesicles in haloarchaea [11,32]. As larger GvpC protein variants were supplied via expression plasmids, we observed generally longer and wider vesicles, suggesting that the nanoparticles were increasingly strengthened. Similar results were also obtained for some cyanobacteria, where a strengthening role for the GvpC protein was reported [19,33]. In one study, *A. flos-aquae* GvpC protein produced in *E. coli* could bind and strengthen the structures after native GvpC protein had been removed by urea treatment. GvpC genes have been reported in most if not all gas vesicle-containing haloarchaea and cyanobacteria, indicating that the protein may serve similar functions in these two groups of aquatic microorganisms. However, *gvp*C is reportedly absent in other *gvp* gene-containing species, a finding suggesting that it may not be absolutely essential for biosynthesis of gas vesicles [1,15].

An interesting feature of the GvpC protein is the presence of internal repeats (8 in *Halobacterium* sp. NRC-1) [9]. Our results show that even a small subset of these repeats in truncated variants of GvpC proteins is sufficient to permit binding to GVNPs. In the pARK-C1 construct, only 3 copies are present, while in pARK-C2, there are 5. Both of these plasmids produced proteins that bound to the vesicles. The longer GvpC variants produced from pARK-C3 and C4 (7 or 8 repeat copies, respectively), complemented production of the nanoparticles considerably better than the smaller GvpC proteins, based on both colony phenotype and vesicle morphology. Similar conclusions were previously reported for *A. flos-aquae* GvpC protein variants containing three or four repeats (out of 5 in the full-length protein) in *in vitro* experiments [34]. In this cyanobacterial system, GvpC depleted vesicles had their strength better restored with proteins containing larger numbers of repeats. In *Halobacterium* sp. NRC-1, the presence of a highly acidic C-terminal region suggests a further role for this feature in stabilizing gas vesicles, likely reflecting the high salinity found in the cytoplasm.

We used a synthetic *Gaussia princeps* luciferase gene to further assess the binding of GvpC fusion proteins to gas vesicles. Initially, we found that the luciferase protein was active when produced in *Halobacterium* via expression vectors alone (our unpublished results) or as a fusion with the GvpC fragments or full-length protein, demonstrating that the marine enzyme was capable of adopting an active structure even after exposure to the hypersaline cytoplasm of *Halobacterium*. Further investigation showed that the GvpC-luciferase fusion proteins were bound to buoyant gas vesicles, confirming that the enzyme is likely displayed on the surface of nanoparticles. Although antigenic proteins and protein fragments have been previously found to be displayed on gas vesicles, these findings now show that an enzyme may also decorate the nanoparticles while retaining its catalytic activity. Moreover, when two different *gvp*C genes (wild-type and shortened/fused to luciferase) were present, we found that both GvpC forms were bound to the nanoparticles. These results extend the possible biotechnological uses of GVNPs to applications requiring multivalency.

All together, our results provide improved genetic and plasmid resources for engineering of GVNPs for biotechnological applications. The original system described required the incorporation of target genes into a large plasmid containing the entire *gvp* gene cluster, pFM104d, and a natural mutant strain deleted for the gene cluster, SD109 [7,8,11-13,35]. The newly described system utilizes the much smaller and more versatile plasmid series, pARK and pDRK, containing a relatively small portion of the *gvp* gene region. The new system allows more facile cloning of genes of interest into the smaller expression vectors and replacement of only a single deleted gene (Δ*gvp*C) in the *gvp* gene cluster. These features will greatly facilitate expression of foreign proteins in GVNPs, including antigenic proteins from pathogenic microorganisms for vaccine development.

## Conclusions

Gas vesicle nanoparticles (GVNPs) in the halophilic archaeon, *Halobacterium* sp. NRC-1, are successfully being used for antigen display and vaccine development. The genetic tools for bioengineering GVNPs have now been greatly improved through construction of a *Halobacterium* strain deleted for the *gvp*C gene and smaller plasmids for expression of foreign proteins fused to GvpC proteins. The utility of the improved system has been demonstrated by expression of an active *Gaussia princeps* luciferase enzyme fused to GvpC and bound to buoyant gas vesicles. These results establish a significantly improved genetic system for displaying foreign proteins on GVNPs and extend the bioengineering potential of these novel nanoparticles to catalytically active enzymes.

## Methods

### Culturing and nanoparticle preparation

*Halobacterium* strains used for this study (Table 1) included NRC-1, the wild-type (ATCC 700922/JCM11081) [28], SD109, with deletion of the entire gas vesicle gene cluster [13,35], SD109 (pFM104*gvp*C::κ1), with insertions of a kanamycin (κ) cassette in the *gvp*C gene [11], NRC-1Δ*ura*3 [24-26] for gene knockouts, and NRC-1Δ*ura*3Δ*gvp*C constructed in this study. These strains were grown in CM^+^ media, as previously described, with the addition of mevinolin (20 μg/ml) (generously provided by Merck, Sharp, and Dohme, Rahway, NJ) when transformed with expression plasmids [26,27].

For preparation of nanoparticles, lawns of *Halobacterium* cells were collected by washing with 5 ml of PBS solution [137 mM NaCl, 2.7 mM KCl, 10 mM sodium phosphate dibasic, and 2 mM potassium phosphate monobasic (pH 7.4)] containing 1.0 mM MgSO_4_. Ten μg/ml of DNase I (Roche Diagnostics, Indianapolis, IN) was added and the cell lysate suspension was incubated for 3 hours at 37°C. Lysates were centrifuged at 60 × g overnight in a swinging bucket rotor using a Jouan CR412 centrifuge (Thermo Scientific, Rockford, IL) to accelerate flotation of the gas-filled nanoparticles. Next, intact buoyant nanoparticles were carefully collected into a clean tube and resuspended in PBS solution, floated by overnight centrifugation, as above, and re-collected. The flotation procedure described above was repeated until a milky white suspension of GVNPs was obtained.

For preparation of whole cell extracts, liquid cultures of *Halobacterium* strains were grown in an illuminated Innova 44 incubator shaker (New Brunswick Scientific, Enfield, CT) at 42°C with shaking at 220 rpm. Ten ml cultures (OD 1.2 at 600 nm) were harvested by centrifugation (8000 rpm × 10 min at 4°C) in a Sorvall RC-5B centrifuge. Pellets were resuspended in 0.5 ml of sterile distilled water containing freshly prepared 1 mM phenylmethylsulfonyl fluoride (Sigma Corporation, St. Louis, MO), 10 μg/ml DNase I was added, and the lysates incubated at 37°C for 30 minutes and dialyzed against 4 liters of distilled water at 4°C overnight. Protein concentrations were determined by the Bradford dye (Bio-Rad Laboratories, Hercules, CA) binding method [36] using bovine serum albumin (BSA, Sigma Corporation) as a standard.

### Construction of *Halobacterium* Δ*gvp*C strain

Approximately 500 bp regions flanking *gvp*C were amplified by crossover PCR (using primers shown in Table 2). The resulting amplified crossover PCR fragment was cloned using flanking *Hin*dIII sites incorporated in the primers, into the *Hin*dIII site of pBB400 (Table 1) [26]. The resulting plasmid, pBB400Δ*gvp*C, deleted 359/382 codons of the internal portion of the *gvp*C gene, but retained the first and last seven codons of *gvp*C as well as seven additional codons in the crossover region. The final construct was sequenced to verify correctness of the inserted PCR fragment and transformed into *Halobacterium* sp. NRC-1Δ*ura*3 using the PEG-EDTA method [37].

**Table 2:** Oligonucleotides used in this study

**Oligonucleotide**	**5′-3′ sequence**	**Use**
*gvp*Cdel5F	CGCAAGCTTATTACTTCTCTCCAGTCGATG	*gvp*C deletion construction
*gvp*Cdel5R	GCGGGCAGTACTCATCTCGTCCTCGAGGCGTTTGTCTGTGACACTCAT	
*gvp*Cdel3F	GACGAGATGAGTACTGCCCGCCGGCCGGATGATAAAACATGA	
*gvp*Cdel3R	CGCAAGCTTACTCGTTGTAGACCAGCGTTG	
*gvp*A-*Nde*I	CTCAAGGTATACCACTAGACCCTAAT	Amplification of *gvp*A promoter
*gvp*A-*Kpn*I	ACTCATGGTACCTACTTCTCTCCAGT	
*gvp*C-F1*Afl*II*Gvp*C-F	GGTGTGCTTAAGATGAGTGTCACAGACAAA	*gvp*C gene segment amplification
*gvp*C-C1R*Avr*II*Gvp*C	CAGCCTAGGGTGGGTTGAGTTCATCTCTGT	
*gvp*C-C2R*Avr*II*Gvp*C	CTGCCTAGGCGCGGTAGCGTCGAAGCTGTC	
*gvp*C-C3R*Avr*II*Gvp*C	GTGCCTAGGTTCTGCTTCCGCTTCGAC	
*gvp*C-C4R*Avr*II*Gvp*C	AGACCTAGGTGTTTTATCATCCGGCCG	
*gvp*C-His-adapter F	CGTCTCCATATGCACCACCACCACCACCACCTTAAGCGTCTACCTAGGAGCGCTTGAGGATCCATC	His-tag adapter for *gvp*C and antigen fusion expression plasmid construction
*gvp*C-His-adapter R	GATGGATCCTCAAGCGCTCCTAGGTAGACGCTTAAGGTGGTGGTGGTGGTGGTGCATATGGAGACG	
pKJ-*csp*D2F	GCTGGACTGCCTTTTCTTCG	Sequencing of promoters and inserts in pARK and pDRK series plasmids
pKJ-*Bam*H1-3′160R	GTTACTCCACCGTCATTCAG	
Universal F 20mer	GTTGTAAAACGACGGCCAGT	Sequencing across *gvp*C deletion
Universal R 20mer	CACAGGAAACAGCTATGACC	
Luci Int R	GTGGCTGAGGCAGATGAGGC	Sequencing and determination of luciferase gene orientation

pBB400Δ*gvp*C transformants were selected by plating on CM^+^ media lacking uracil (HURA), colonies picked and grown in liquid HURA media, and genomic DNA extracted, as previously described [26,37]. Integrant candidates were screened by PCR using the flanking primers and genomic DNA as template, and integrants were plated on CM^+^ plates containing 250 μg/ml 5-fluorouracil (5-FOA) (Toronto Research Chemicals, North York, Canada). Excisant colonies were picked and grown in liquid CM^+^ media containing 5-FOA, genomic DNA was extracted, and PCR reactions were used to screen for knockout mutants using primers flanking the *gvp*C gene (Table 2).

### Construction of the pARK and pDRK expression plasmids

For construction of the *Halobacterium* sp. pARK and pDRK expression plasmids, pMC2 expression plasmid was used as the backbone [26,27]. The β-galactosidase gene was excised and replaced with an adapter (see Table 2) containing a start codon, hexahistidine-tag (His-tag), and *Afl*II, *Avr*II, and *Afe*I restriction sites. The C1-C4 GvpC fragments were PCR amplified and inserted via the *Afl*II and *Avr*II sites, and the synthetic *Gaussia princeps* luciferase gene (LifeTechnologies, Grand Island, NY) was inserted via the *Afe*I site [23]. The promoter region was replaced via the *Kpn*I and *Nde*I sites. The constructs were validated by DNA sequencing.

### Electron microscopy

For thin-sectioning, cells were fixed in 3% glutaraldehyde-20% NaCl, postfixed in 2% OsO_4_-20% NaCl for 4 hours, rinsed with 20% NaCl, stained with 5% uranyl acetate in 20% NaCl-20% acetone for 1 hour, and then dehydrated by immersion in a series of isotonic acetone solutions. Samples were then embedded in Spurr medium which was polymerized at 70°C for 8 hours [38]. Thin sections of 600 Å (60 nm) were examined on copper grids stained with lead [39].

For negative staining, purified nanoparticles were adsorbed to glow discharged 400 mesh carbon coated parlodion copper grids for 30 seconds. Grids were then rinsed in distilled deionized water, 3 times for 30 seconds each. Nanoparticles were negatively stained two times for 30 seconds each in 1% uranyl acetate with 0.04% tylose. Grids were blot dried with Whatman #1 filter paper and samples imaged on a Hitachi 7600 TEM at 80 kV. Images were captured with an AMT CCD (1 K × 1 K) camera at 8,000× and 30,000× magnifications. Fifty representative gas vesicle nanoparticles from each strain were measured and average values and standard deviations calculated.

### Western blotting analysis

The methods used were similar to those previously described [15]. Briefly, cell lysates containing 50 μg of protein or purified gas vesicle nanoparticle preparations containing 2 μg of protein were electrophoresed on 12% polyacrylamide-SDS gels, for 90 minutes at 100 volts using a Bio-Rad vertical gel electrophoresis unit. Proteins were transferred to 0.45 μm Immobilon-P polyvinylidene difluoride (PVDF) membranes (Millipore Corp., Boston, MA) for 1 hour at 100 volts using a Bio-Rad gel blotter. The membranes were washed twice for 5 minutes with TBS buffer [20 mM Tris–HCl (pH 7.6), 137 mM NaCl], blocked for 1 hour with 5% BSA in TBS buffer, incubated overnight at 4°C with affinity column purified rabbit GvpC antibodies (Thermo Scientific) diluted 1:500 [15] or rabbit anti-His-tag antibody (Cell Signaling Technology, Beverly, MA) diluted 1:750. Membranes were then washed 5 times each for 5 minutes with TBS buffer containing 0.1% Tween 20, and incubated with goat anti-rabbit secondary antibodies labeled with alkaline phosphatase (Sigma Corporation), diluted (1:2500) in a solution containing 5% BSA in TBS buffer. For detection of the protein bands, the membrane was incubated in 1-Step NBT/BCIP Substrate (Thermo Scientific) according to the manufacturer’s specification.

### Luciferase activity

Whole cell lysates or purified gas vesicle nanoparticles prepared as described above were assayed for *Gaussia princeps* luciferase activity using the Glow Assay system (Thermo Scientific) according to the manufacturer’s specification. Assays were conducted in 96-well plates using a SpectraMax M5 luminometer (Molecular Devices, Sunnyvale, CA). Induction was calculated in relative light units of the treated sample/average relative light units of the untreated samples.

## Competing interests

The authors declare that they have no competing interests.

## Authors’ contributions

SD designed the study and wrote the manuscript. RK conducted Western and protein analysis and carried out cloning and molecular biology, PD assisted with manuscript preparation, bioinformatics, cloning and molecular biology, and gas vesicle microscopy, SB assisted with molecular biology and gas vesicle microscopic analysis, FE assisted with microbiology and gas vesicle microscopic analysis, and BS conducted the electron microscopy. All authors read and approved the final manuscript.
